# Influence of Housing Conditions on Reliability of Immunocastration and Consequences for Growth Performance of Male Pigs

**DOI:** 10.3390/ani10010027

**Published:** 2019-12-21

**Authors:** Kevin Kress, Ulrike Weiler, Sonja Schmucker, Marjeta Čandek-Potokar, Milka Vrecl, Gregor Fazarinc, Martin Škrlep, Nina Batorek-Lukač, Volker Stefanski

**Affiliations:** 1 Department of Behavioral Physiology of Livestock, Institute of Animal Science, University of Hohenheim, Garbenstraße 17, 70599 Stuttgart, Germany; weiler@uni-hohenheim.de (U.W.); sonja.schmucker@uni-hohenheim.de (S.S.); volker.stefanski@uni-hohenheim.de (V.S.); 2 KIS–Agricultural Institute of Slovenia, Hacquetova ulica 17, 1000 Ljubljana, Slovenia; meta.candek-potokar@kis.si (M.Č.-P.); martin.skrlep@kis.si (M.Š.); nina.batorek@kis.si (N.B.-L.); 3 Veterinary Faculty, Institute of Preclinical Sciences, University of Ljubljana, Gerbičeva 60, 1000 Ljubljana, Slovenia; milka.vrecl@vf.uni-lj.si (M.V.); gregor.fazarinc@vf.uni-lj.si (G.F.)

**Keywords:** immunocastration, vaccination, Improvac, non-responder, immune response, housing conditions, surgical castration, boar taint, growth performance, genital tract

## Abstract

**Simple Summary:**

Surgical castration of male piglets is societally criticized as it is painful and violates the integrity of the animals. Pork production with boars and immunocastrates are possible alternatives. Even if immunocastration is an animal-welfare-friendly alternative, its market share is low and the reliability of this technique is discussed controversially within the pork chain. Currently, the number and the reason for non-responders to vaccination are not clear. Various factors may contribute to impaired immune response including adverse and stressful housing conditions. This study, therefore, examines the influence of different housing conditions on the immune response after two Improvac^®^ vaccinations. To determine vaccination success, testosterone concentrations, GnRH-binding, and boar taint compounds were evaluated. Furthermore, the growth performance of male pigs was compared. The results show that immunocastration is reliable under different housing systems and prevents boar taint. Moreover, the growth performance of immunocastrates is high and even superior to that of boars and barrows after the 2nd vaccination. Accordingly, immunocastration is not only animal-welfare-friendly but also economically attractive and suitable for different housing systems.

**Abstract:**

Immunocastration is a sustainable alternative to piglet castration but faces limited market acceptance. The phenomenon of non-responders has not to date been examined in detail, but adverse and stressful housing conditions (e.g., mixing of groups) might impair the success of vaccinations. Therefore, we evaluated the influence of housing conditions on the immune response after two Improvac^®^ vaccinations at an age of 12 and 22 weeks, respectively. Boars, immunocastrates and barrows (*n* = 48 each) were assigned to three different housing conditions (*n* = 36 enriched, *n* = 36 standard *n* = 72 repeated social mixing). Immune response was quantified by measuring GnRH-binding and its consequences for testosterone concentrations, development of the genital tract and boar taint. Growth performance was evaluated via average daily gain (ADG). GnRH-binding and testosterone levels revealed that immunocastration reliably suppressed testicular functions after the 2nd vaccination. Housing conditions did not modify testicular function but influenced ADG as animals under mixing grew slower than those under enriched conditions. Gonadal status had only a slight impact on ADG except in immunocastrates, which showed a temporarily higher ADG after the 2nd vaccination. The results show that immunocastration is a reliable procedure under different housing conditions and competitive in terms of growth performance.

## 1. Introduction

Traditionally, male piglets designated for pork production have been surgically castrated within the first week of life to avoid boar taint and to prevent problems due to male-specific behavior [1]. As the surgical castration is mostly carried out without anesthesia and analgesia, the animals suffer due to the pain inflicted on them as a consequence of the surgical castration [2,3]. As a consequence, this technique is facing increasing societal criticism [4]. In 2010, these circumstances led European stakeholders of the pork chain to commit themselves voluntarily to stop surgical castration of male piglets by 2018 [5]. Today, however, about 60% of all male piglets produced in Europe are still surgically castrated [6] as pork production with boars still faces problems due to boar taint and other meat quality and animal welfare issues [7]. Quality problems result from the accumulation of the two main boar taint compounds, androstenone and skatole, in adipose tissue [8]. Furthermore, boar carcasses are leaner than those of barrows and have softer adipose tissue due to higher amounts of polyunsaturated fatty acids, which reduce their suitability for processing especially in the case of high-quality dry meat products [9]. From an animal welfare point of view, pork production with boars may lead to boar-specific welfare problems related to sexual behavior and aggression (e.g., injuries, lameness) due to boars’ higher potential for agonistic behavior [10,11].

An alternative to both surgical castration and pork production with boars is immunocastration. Immunocastration is an active immunization against GnRH, a key hormone regulating testicular functions. The treatment consists of two consecutive vaccinations that trigger an immune response which results in the production of antibodies against the endogenous hypothalamic hormone GnRH [12]. Until the second vaccination, immunocastrates are from a physiological perspective similar to boars, with the same anabolic potential but also with the same welfare-associated problems. After the second vaccination, testicular hormone synthesis ceases and boar-specific behavioral problems decline within two weeks [13] as do the number of injuries (e.g., penile injuries) [14]. In Europe, only one vaccine (Improvac^®^) is currently available for commercial use [13]. Although Improvac^®^ has been approved by the EMA (European Medicines Agency) in the European Union since 2009 [15], the market share of immunocastrates in Europe is only about 2.8% to date [6]. The reasons for the low market acceptance of this technique are diverse and are mainly due to knowledge gaps regarding the optimal use of immunocastration for various market demands. In order to expand the market share of immunocastrates, vaccination has to work reliably under different production systems, and the growth performance of immunocastrates has to be competitive [13]. However, some reports describe a lower vaccination reliability, with about 0%–3% of vaccinated animals responding poorly to the vaccinations. These are the so-called “non-responders”. The reasons for poor immune response are not clear and may be due to either accidentally missed vaccinations or due to health problems in animals during the vaccinations [9,13,16,17,18].

Another possible explanation might be stress-induced immunosuppression. Studies in humans and animals have amply shown that social stressors can impair the immune system [19,20]. Antibody response, for example, was found to be suppressed in defeated rats [21] or highly stressed human caregivers in response to influenza virus vaccination [22]. As stress can have a negative impact on the porcine immune system [23], stressful housing conditions might be a predisposing factor for non-responders. In pork production, unstable social environments cannot be avoided and social mixing is a common procedure (e.g., at the beginning of the fattening phase, while sorting into homogeneous groups etc.). It is well known that social mixing and unstable groups lead to social stress in pigs [24], with more aggressive behavior [25,26] and a negative impact on the pigs’ immune system [26,27]. Social mixing not only temporarily activates the hypothalamic–pituitary–adrenal (HPA) axis by increasing cortisol levels after a mixing event has occurred [25,27], but may also lead to chronic stress if frequent mixing persists over a longer period of time [25]. Alternative housing systems such as outdoor housing or organic farming can have both positive and negative effects on the welfare and health status of pigs. In alternative housing systems, pigs may be able to behave in a species-specific way with pen partners, but on the other hand are at an increased risk for exogenous factors such as sunburn or ecto- and endoparasites [28]. A study by de Groot et al. [29] showed that pigs housed under enriched housing conditions had higher salivary cortisol levels than pigs housed under barren housing conditions, whereas no differences existed in immune parameters such as proliferation of leukocytes or lymphocytes. Thus, housing conditions had no clear impact on the immune system. The formation of antibodies against a vaccine was however not studied. These results therefore do not exclude the possibility that a moderate impairment of animals’ antibody response due to housing conditions increase the risk for occurrence of non-responders to immunocastration. Social stress not only affects the pigs’ immune system but also their growth performance, which is lower in pigs exposed to social stress and mixing [30,31,32].

The aim of this study was to investigate whether housing conditions have an impact on the immune response after two Improvac^®^ vaccinations against GnRH in male pigs, and on the creation of non-responders. The influence of housing conditions and castration status of male pigs on growth performance during the fattening period was also investigated.

## 2. Materials and Methods

### 2.1. Animals and Experimental Setup

The experimental protocol was approved by the ethical committee for animal experiments by the regional authority of Tuebingen, Germany, (ID HOH 47/17TH), and all procedures were conducted in accordance with the German Animal Welfare Act. In total, two subsequent trials were conducted with 72 pigs per trial. The trials were performed at the animal experimental unit of the University of Hohenheim (Unterer Lindenhof, Eningen, Germany). All pigs were a crossbreed of Pietrain x German Landrace. In this experiment, three different sex groups of male pigs (boars, *n* = 48; immunocastrates, *n* = 48 and barrows, *n* = 48) were housed under three different housing conditions (standard: *n* = 36; enriched: *n* = 36; mixing: *n* = 72). In the ‘standard’ scenario, the animals were housed in conventional housing conditions (1, 2 m^2^ per pig). Under ‘enriched’ conditions, the animals had twice as much space (2, 6 m^2^ per pig) as under ‘standard’ conditions and additional access to the outdoor area (3, 1 m^2^ per pig). In the ‘mixing’ scenario, the animals were kept similar to ‘standard’ conditions, but the groups were mixed repeatedly to induce social stress. Mixing consisted of an exchange of two of 6 animals per pen with two unfamiliar animals from another pen of a similar sex group every third day of the mixing phase. For this reason, the animal number of the mixing scenario was twice as high as in the two other housing conditions. Mixing was assigned around vaccination time points to maximize probable effects of social stress on vaccination outcome. Thus, mixing started 7 days before the first vaccination at an age of 11 weeks, with a total number of 5 mixing events. The second mixing phase started at an age of 20 weeks and consisted of 8 mixing events over 24 days. The selection of animals which were mixed was randomized.

**Figure 1:**
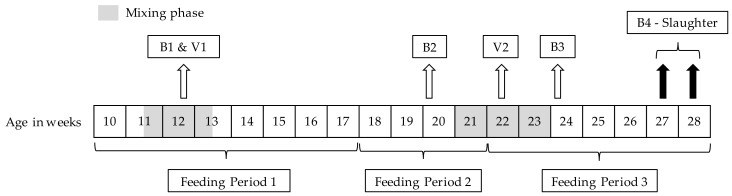
Generalized timeline of the trials (feeding periods, blood samples (B1–B4), vaccination times (V1—applied immediately after B1, V2), mixing periods, and slaughter dates according to the age (weeks) of the animals).

The animals for this experiment had been selected from a total of 48 litters (322 male piglets). The piglets were allocated randomly at an age of three days to 9 different experimental groups (sex group x housing) by the method of Latin Squares. Barrows were surgically castrated within the first week of life without anesthesia, but received 0.2 mL Metacam^®^ (Meloxicam, 5 mg/mL) as post-surgery pain relief. Immunocastrates (IC) were vaccinated twice with Improvac^®^ at an age of 12 (first vaccination—V1) and 22 weeks (second vaccination—V2) as shown in the timeline of the experiment in Figure 1. It was decided that full siblings were not to be assigned to the same interaction of sex group x housing condition. The assignment of the interaction of the sex group x housing condition to the respective pens was also randomized, and it was ensured that two mixing groups of the same sex group (e.g., boars) were not located directly next to each other.

In all pens, chopped straw (500 g per pen) and sawdust (1000 g per pen) were supplied daily. Feed was supplied twice per day between 7:45 am and 8:00 am and between 4:00 pm and 4:30 pm. The pigs were fed ad libitum. Feed composition varied during the fattening period (three phase feeding) and was based on the recommendations for intact boars (see Kress and Verhaagh [33]). In order to avoid a feeding effect, all animals were fed the same diet. Individual weights of all animals were recorded at birth, at an age of 3 weeks, 10 weeks, 17 weeks and at slaughter to characterize the growth performance via average daily gain (ADG).

The experiment covered the period between an age of 10 weeks and 27 or 28 weeks (slaughter). Due to the limited capacity of the experimental slaughter unit (LSZ Boxberg, Boxberg, Germany), the animals were slaughtered on two different occasions to ensure standardized conditions and complete data collection. 

In total, 4 blood samples were collected from each pig to measure antibody titer against GnRH, as well as testosterone and cortisol levels at an age of 12 weeks (B1, to analyze differences between sex groups and housing conditions immediately before V1), 20 weeks (B2, after V1 and before V2, to measure the immune response after V1 and the impact of sex group on testosterone concentrations) and 24 weeks (B3, two weeks after V2 and immediately after the second mixing phase to analyze the antibody response after V2 and the corresponding testosterone concentrations and to analyze the impact of social mixing on cortisol concentrations) and at slaughter line (B4, to measure the final antibody titer against GnRH and testosterone concentrations at slaughter to confirm that testicular functions are suppressed until slaughter and to analyze differences in sex groups and housing conditions on transport and slaughter stress) (as given in Figure 1). For blood sampling during the experiment, the animals were separated individually and fixed by a snare pole and blood was collected by puncture of the *vena jugularis externa* into heparinized vials. Plasma was removed after centrifugation and stored at −20 °C until further analyzed.

### 2.2. GnRH Binding in Plasma

Success of immunocastration was assessed by measuring GnRH binding in plasma with an in-house assay, based on ^125^I-GnRH. GnRH-Iodination was carried out with the solid phase Iodogen-method according to Salacinski et al. [34], using 1 µg Iodogen/cup, 200 µCi ^125^I (Na^125^I, Hartmann Analytik GmbH, Braunschweig, I-RB-31.) and 200 ng GnRH (Fisher Scientific, PEP-168) diluted in 0.5 M phosphate buffer (pH 7,4). After an incubation period of 3 min the free iodine was separated from the iodinated peptide with an anion-exchange resin column. The specific activity was about 200 nCi/ng GnRH. In order to determine the GnRH binding, 15,000 cpm ^125^I-GnRH (corresponding to 17.5 pg GnRH) in 100 µl in 0.1 M phosphate buffer were incubated with 5 µl of plasma and 200 µl of 0.1 M phosphate buffer with the addition of bovine serum albumin (BSA, 0.1%) at 4 °C for 24 h. Afterwards, bound free separation was carried out with dextran-coated charcoal (0.5%) in 1 mL H_2_0 and subsequent centrifugation. The supernatant was counted for one minute in a gamma counter. As controls, a pool sample of vaccinated animals with a good response (pool A) and a pool sample of non-vaccinated boars (pool B) were measured within each assay. The absolute binding of the biological samples was calculated (counts/total counts). The specific binding of pool A was 39.38% ± 6.29%; (CV: 16%; range 35.16% to 61.02%) in trial 1, and 38.79% ± 2.66%; (CV: 7%; range 39.22% to 56.74%) in trial 2. The non-specific binding determined with pool B was 4.44% ± 1.02%, (CV: 23%; range 1.35% to 2.67%) in trial 1, and 5.65% ± 0.76% (CV: 13%; range 4.20% to 6.33%) in trial 2. 

### 2.3. Testosterone Levels in Plasma

Testosterone concentrations in plasma were determined in duplicate with a direct in-house radioimmunoassay (RIA). In brief, 20 µL plasma were incubated with [1,2,6,7-^3^H]- testosterone (95.5 Ci/mmol, PerkinElmer, Boston, MA, USA) and antiserum. The antiserum had been raised in a rabbit against testosterone-3CMO-BSA and was used at a final dilution of 1:144,000. Cross reactivity was 67% with 5αDHT, and below 2% for other tested steroids. Charcoal-treated plasma (20 µL) was added to the calibration curve to compensate for substrate effects in case of measurements in plasma. Bound free separation was carried out with 0.5 mL ice cold solution of dextran coated charcoal (0.5%) in H_2_0 and subsequent centrifugation. The supernatant was transferred into counting vials with scintillation fluid and counted in a beta-counter. To determine the precision of the tests, plasma samples from barrows were spiked with defined concentrations of 0.5 to 10.0 ng/mL (precision 100%–125% recovery in each trial). In addition, biological samples were included to determine the repeatability of the measurements (coefficient of variation: intra-assay 1.99% (trial 1) and 5.22% (trial 2); inter-assay 8.46% (trial 1) and 6.87% (trial 2)).

### 2.4. Cortisol Levels in Plasma

In order to determine the cortisol concentrations of the respective experimental animals, a radioimmunoassay (RIA) was carried out as described by Engert et al. [35]. A polyclonal antibody against cortisol-3-BSA (MBS316242, MyBioSource, San Diego, CA, USA) at a final dilution of 1:112,000 in 0.1% BSA buffer was added and as a tracer [1,2,6,7-3H] cortisol (93 Ci/mmol, PerkinElmer, Boston, MA, USA) used. All samples of each animal were measured in a single assay. Intra-assay variance for a biological sample was 4.87% and inter-assay variance was 8.87%.

### 2.5. Boar Taint Compounds in Adipose Tissue

For the determination of boar taint compounds, samples of subcutaneous fat were vacuum packed at slaughter and stored at −20 °C until the start of the analyses (within 14 days after sampling). Androstenone and skatole concentrations were determined with high-performance liquid chromatography (HPLC, HP 1200, Agilent Technologies, Waldbronn, Germany) equipped with a fluorescence detector according to Pauly et al. [36]. Adipose tissue samples (10–20 g) were put in a microwave oven for 2 × 1 min at 350 W. Afterwards, the liquefied lipid fraction was removed and centrifuged for 20 min at 11,200 g and ambient temperature. After centrifugation, fat was heated to 50 °C and 0.5 ± 0.01 g water-free liquid fat transferred into 2 mL Eppendorf tubes adding 1 mL of internal standards diluted in methanol (0.496 mg/L androstenone and 0.050 mg/L 2-methylindol for androstenone and skatole determination, respectively). After stirring for 30 s, the tubes were incubated for 5 min at 30 °C in an ultrasonic water bath, kept on ice for 20 min and centrifuged for 20 min at 11,200 g at 4 °C. For androstenone determination, 50 µL of the supernatant was submitted to derivatization with dansylhydrazine and boron trifluoride (BF3) for 2 min. An aliquot of 10 µL of the derived mixture was then injected into the column (SunFire C18 3.5 μm 4.6 × 75 mm equipped with 20 mm precolumn) and analyzed using fluorescence (at λex = 346 nm, λem = 521 nm). For skatole determination, 20 µL of the supernatant was injected into the column and analyzed using fluorescence (λex = 285 nm, λem = 340 nm). Concentrations were expressed per g of the liquid fat. The detection limits were 0.24 μg/g for androstenone and 0.03 μg/g for skatole. For androstenone concentrations below detection limit, a value of 0.21 μg/g was assumed, and for skatole concentrations of 0.02 μg/g (half of lowest value). Inter- and intra-assay variation for both compounds was below 10%. Carcasses were classified with a threshold for androstenone of 1 µg/g fat and skatole of 0.25 µg/g fat [33].

### 2.6. Genital Tract Measurements

The efficacy of immunocastration was further assessed by genital tract measurements as follows: reproductive organs/accessory sex glands and the pelvic part of the urogenital tract were excised and weighed at slaughter line as described by Fazarinc [37]. For this purpose, the pelvic part of the urogenital tract was first separated from the rectum and anus and the urinary bladder emptied through an incision at its apex. The pelvic urogenital tract was then cleaned of excessive adipose and connective tissue and the penis removed by cutting off close to the caudal end of the bulbourethral glands. The dissected pelvic urogenital tract consisting of the accessory reproductive glands (i.e., paired vesicular and bulbourethral glands, and prostate) was then weighed. Subsequently, accessory reproductive glands and testes with epididymes from each boar and immunocastrate were dissected and weighed individually. 

### 2.7. Statistical Analysis

Data were analyzed with SAS Version 9.4 (SAS Institute Inc., Cary, NC, USA), using a linear mixed model of the MIXED (mixed linear model) procedure with degrees of freedom determined by the method of Kenward-Roger. Variance components were estimated using the restricted maximum likelihood (REML) method. The linear mixed model included sex group, housing conditions and the interaction of sex group x housing as fixed effect. As the interaction of sex group x housing condition was mainly insignificant (except in two cases, skatole and cortisol B3), only *p*-values of sex group and housing conditions are presented in the tables.

Furthermore, trial, pen and the interaction of trial x pen, slaughter date, dam, sire and the interaction of dam x sire were used as random effects. Residuals were tested on normal distribution and variance homogeneity by visual check of residuals plots [38]. If the residuals were not normally distributed (androstenone and skatole), the data were logarithmically transformed and the results then retransformed. Differences between groups were adjusted by a Bonferroni correction. Paired Student’s *t*-tests (two-tailed) with Bonferroni correction were used to analyze differences between a priori specified blood samples (B1 vs. B2; B2 vs. B3; B3 vs. B4) within one sex group with SPSS Version 24 (IBM Corp., Armonk, NY, USA). *p*-values with *p* < 0.05 were considered as significant and *p* < 0.10 as a tendency. The results are presented as LS-means (last mean square) ± SEM (standard error of the mean).

## 3. Results

### 3.1. Characterization of Testicular Functions in Male Pigs

**Figure 2:**
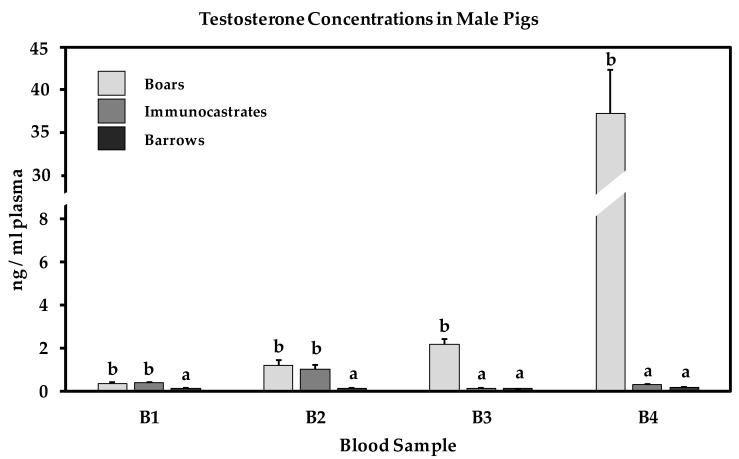
Plasma testosterone concentrations of boars, immunocastrates and barrows (*n* = 48 per sex group) at four blood sample time points (B1: before V1, B2: before V2, B3: 2 weeks after V2, B4 at slaughter). Testosterone concentrations between different sex groups with different superscripts differ significantly (*p* < 0.05).

Mixed linear model analysis indicated that sex group had a significant impact on testosterone concentrations, whereas housing conditions and the interaction of sex group and housing condition did not modify testicular functions. Figure 2 shows plasma testosterone concentrations of boars, immunocastrates and barrows during blood sampling (B1–B4) throughout the fattening period. The first blood sample B1, collected before V1, revealed similar testosterone concentrations in boars and immunocastrates (about 0.3 ng/mL). Both sex groups, however, had higher testosterone levels than barrows (about 0.13 ng/mL). Using Bonferroni corrected *t*-tests, changes in testosterone concentration were further analyzed in detail within each sex group. Results indicate that in barrows, testosterone levels remained at low levels between B1–B3, but were slightly higher at B4 (*p* < 0.001). In contrast, testosterone concentrations in boars increased during the fattening period and reached considerably high levels at slaughter (about 39 ng/mL). Testosterone concentrations in boars differed significantly (*p* < 0.01) between all three specified comparisons (B1 vs. B2; B2 vs. B3; B3 vs. B4). Immunocastrates had similar testosterone concentrations as boars until B2 (V2). Two weeks after V2 (B3), testosterone concentrations dropped to pre-vaccination levels, similar to that of barrows (about 0.11 ng/mL). At slaughter (B4), testosterone levels in immunocastrates tended to be marginally higher than in barrows (immunocastrates: 0.3 ng/mL vs barrows: 0.2 ng/mL; *p* = 0.056), but substantially lower than in boars (*p* < 0.001). This indicates that immunocastration successfully suppressed testicular functions. In immunocastrates, testosterone concentrations differed between all specified comparisons (*p* < 0.001).

### 3.2. GnRH Antibody Formation and Testicular Functions in Immunocastrates during the Investigation Period

**Figure 3:**
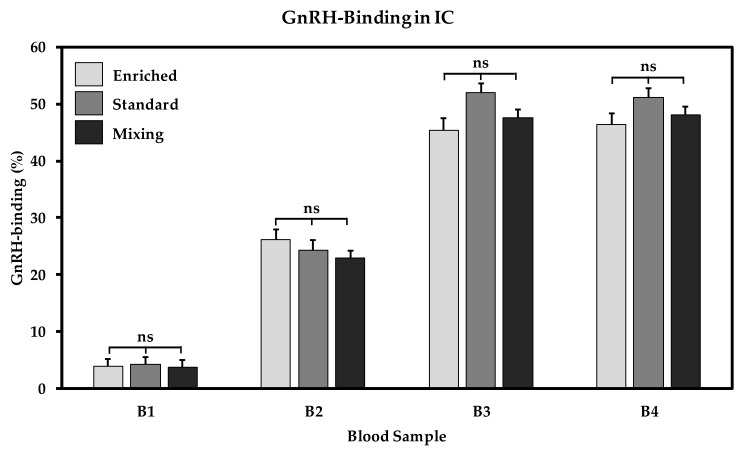
GnRH-binding in immunocastrates under three different housing conditions (enriched—*n* = 12, standard—*n* = 12 and mixing—*n* = 24) at four blood sample time points (B1: before V1, B2: before V2, B3: 2 weeks after V2, B4: at slaughter). Differences between housing groups marked with ns are not significant.

In Figure 3, GnRH-binding of immunocastrates is given for the four blood samplings (B1–B4). Before V1 (B1), all immunocastrates revealed low unspecific GnRH-binding which corresponds to the low, unspecific binding of boars (B1: 3.71 ± 1.31%; B2: 3.04 ± 1.05%; B3: 2.63 ± 1.21%; B4: 2.58 ± 1.17%) and barrows (B1: 3.54 ± 1.32%; B2: 3.13 ± 1.03%; B3: 3.94 ± 1.31%; B4: 3.64 ± 1.24%). In immunocastrates, 2 weeks after V1, GnRH-binding increased markedly (B2, *p* < 0.001), followed by a further increase after V2 (B3, *p* < 0.001). This high GnRH-binding was maintained in all immunocastrates until B4 (B3 vs. B4, *p* = 0.478). Housing conditions had no significant influence on the level of antibody formation against GnRH at any time of sampling.

### 3.3. Evaluation of Reproductive Organs in Male Pigs

Furthermore, the differences between boars and immunocastrates in the characterization of genital tract weights were used to evaluate the efficacy of immunocastration (Table 1). In all the parameters tested (weight of testes with epididymes, vesicular glands, bulbourethral glands, prostate and pelvic part of the urogenital tract), differences between boars and immunocastrates were significant, which shows that immunocastration induced a regression of reproductive organs compared to boars. Similar to other parameters, housing conditions had no impact on the weight of reproductive organs.

### 3.4. Influence of Treatment and Housing Conditions on Boar Taint

Significant differences in boar taint compounds occurred between sex groups (Table 2). All immunocastrates had androstenone levels below the limit of detection (<0.24 µg/g fat) and consequently below the threshold of 1 µg/g fat. Compared to immunocastrates, 79.17% of all boars had androstenone levels above 1 µg/g fat. Housing conditions had no effect on androstenone levels in boars and immunocastrates. Androstenone is testis-derived and was not analyzed in barrows.

The differences in skatole levels were also significant between sex groups. All immunocastrates and barrows had skatole concentrations below 0.25 µg/g fat. In contrast to immunocastrates and barrows, 6.25% of boars (3 out of 48 animals) had skatole levels above the threshold of 0.25 µg/g fat. All 3 boars with increased skatole levels had concomitant androstenone levels above 1 µg/g fat. While housing conditions had no influence on the skatole values, sex group x housing condition (*p* = 0.0293) had an influence on skatole levels with boars housed under enriched conditions exhibiting lower skatole levels (0.023 ± 0.005 µg/g fat) than boars housed under standard conditions (0.063 ± 0.013 µg/g fat; *p* = 0.0105). It can be concluded that immunocastration was effective in preventing boar taint, and that housing conditions had no modifying influence on boar taint compounds in immunocastrates and barrows, whereas in boars, enriched conditions significantly reduced skatole levels.

### 3.5. Cortisol Levels in Male Pigs

Table 3 shows plasma cortisol concentrations of boars, immunocastrates and barrows throughout the fattening period at B1–B4. Cortisol levels did not differ between sex groups and housing conditions in the first two blood samples. At B3, the interaction of sex group and housing condition was significant (*p* = 0.0405), with no significant differences found in post-hoc testing.

Differences between sex groups were, however, evident at slaughter (B4). Boars had higher cortisol concentrations than immunocastrates (*p* < 0.01), and a tendency towards higher cortisol concentrations than barrows (*p* < 0.1). Immunocastrates and barrows did not differ significantly (*p* = 0.899). Within all sex groups, cortisol concentrations increased significantly from B3 to B4 (*p* < 0.001), which clearly shows the influence of slaughter and transport stress on cortisol levels in male pigs. There was no effect of housing conditions on cortisol concentrations at B4.

### 3.6. Growth Performance of Male Pigs

Growth performances of the three sex groups varied throughout the fattening period (Table 4). At the beginning of the fattening period (feeding phase 1), barrows showed a tendency towards higher ADG than boars (911 g vs. 854 g respectively; *p* = 0.0569). Immunocastrates were between boars and barrows in their growth performance in this period. In the second feeding phase, no differences between the sex groups were obvious. Growth performance of immunocastrates changed after V2, in feeding phase 3, and reached the highest ADG (967 g) of all sex groups. Growth performance was significantly higher in boars than in barrows in feeding phase 3 (*p* = 0.0262). Over the entire fattening period, differences between sex groups were less pronounced and did not reach the level of significance (*p* = 0.069). 

In contrast to other parameters, housing conditions had a significant impact on the growth performance of pigs. Animals from the mixing group revealed lower growth rates in the second and third feeding phase than animals from the enriched group, whereas animals from the standard group were in between. In the second feeding phase, animals from the enriched group had an 8% higher growth rate than animals from the mixing group. In the third feeding phase, the ‘enriched’ animals also had a 6% better growth performance than animals from the mixing group.

## 4. Discussion

Immunocastration and pork production with boars are possible alternatives to surgical castration of male piglets [13]. Pork production with boars has advantages when compared to pork production with barrows, as the feed conversion ratio (FCR) and growth performance are improved [7]. On the other hand, the risk of animal welfare-related problems is increased, as boars have a higher potential for agonistic behavior [13]. In addition, the quality of meat from boars is lower because of boar taint, a reduced intramuscular fat content, and increased amounts of unsaturated fatty acids in the fat which limits its suitability for traditional dry cured meat products [39]. From a scientific point of view, immunocastration has the potential to reduce these problems markedly, but the market relevance of immunocastrates is globally very low and the reliability of this procedure is often questioned as knowledge gaps exist on the market [13]. Therefore, the study analyzed the reliability of immunocastration in different housing conditions and compared the growth performance of immunocastrates with boars and barrows to evaluate whether the technique is competitive. To our best knowledge, this is the first study to test vaccination against GnRH with Improvac^®^ under various housing conditions in experimental trials, measuring antibody response on the basis of GnRH binding and testosterone concentrations. Full siblings were allocated to different sex groups and housing conditions in order to reduce variability due to age and genotype.

In contrast to the literature reports [9,13,17] and the concerns of pork chain actors, our study found no evidence for non-responders. In fact, after two Improvac^®^ vaccinations, the immune response was sufficient in all immunocastrates to fully suppress testicular functions. Zeng et al. [16] described that health problems during the vaccinations were linked to an insufficient immune response to Improvac^®^ vaccinations, and thus resulted in non-responders. In the literature [9,13,17], wrong handling or missed vaccinations are often assumed to be the reasons for non-responders. In the present experiment, we ensured that the animals were healthy during the vaccinations and correct handling and careful vaccinations were ensured by experienced veterinarians. We can therefore conclude from this experiment that if vaccinations against GnRH (Improvac^®^) are carried out correctly, the technique is reliable even under more challenging housing conditions.

Testosterone concentrations in all immunocastrates decreased to the level of barrows after the second immunization against GnRH and remained at this basal level until slaughter. In comparison, intact boars had testosterone levels of 3–5 ng/mL plasma at this age, as similarly described by Zamaratskaia et al. [40]. These effects clearly show, that testicular functions were successfully suppressed in all immunocastrates after two vaccinations with Improvac^®^. In our study, boars revealed considerable testosterone concentrations at slaughter. A previous study by Wesoly et al. [41] describes the influence of transport time on the testicular functions of boars. Testosterone concentrations were increased by 2.2 ng/mL plasma per hour transport time, which shows an impact of pre-slaughter conditions on testicular functions in boars. In this study the transport time from the animal experimental unit to the slaughterhouse was about 3 h, the pre-unloading time about 1 h, followed by another hour until the pigs were actually slaughtered.

In the present study, immunocastration was also effective in the prevention of boar taint. Androstenone levels were below the limit of detection in all immunocastrates, all skatole levels below the defined thresholds for skatole (0.25 µg/g fat), indicating that immunocastration is also reliable in preventing boar taint. Skatole concentrations in barrows and immunocastrates were significantly lower than in boars, as high androstenone, testosterone and estradiol levels in boars inhibit the activity of hepatic skatole-degrading enzymes CYP2E1 and CYP2A [42,43,44,45]. This also agrees with the meta-analysis by Batorek et al. [46] and Nautrup et al. [47]. In our study, housing conditions had no effect on either androstenone nor or skatole levels. However, the opposite was shown in a study by Škrlep et al. [48] in which individually housed animals had lower skatole levels than group-housed animals, but no differences occurred in androstenone concentrations. Furthermore, a higher stocking density resulted in higher skatole levels but lower androstenone levels than in animals housed in a lower stocking density and slaughtered at higher ages. 

In comparison to boars, all reproductive organs of immunocastrates were significantly lighter. These findings are in full agreement with several studies [49,50,51] which show a significant impact of treatment with Improvac^®^ on the development of the male genital tract. The different compartments, however, were affected to a various degree. Above all, the *glandula vesicularis*, which is known to reflect testosterone levels in size and secretory activity [52], might be suggested as an additional parameter for determining the success of the vaccination [50]. However, for practical reasons it is difficult to excise the vesicular gland at slaughter line and to determine its weight. Even if testes weight differs significantly between boars and immunocastrates, this parameter is not recommended for detection of non-responders at slaughter line as there is no clear cut between the testes weight of boars and immunocastrates [53]. Therefore, we do not recommend this parameter to determine the success of an adequate immune response after immunocastration.

Similar to this study, previous reports in pigs showed that challenging housing conditions such as mixing must not necessarily lead to a pronounced influence on the plasma cortisol concentrations in pigs [26,32]. Sutherland et al. [30] found even lower cortisol concentrations in mixed than in control pigs. Notably, animals from enriched (and presumably less stressful) environments also show higher cortisol concentrations during daytime than animals from barren housing conditions [29]. However, it has to be considered that single cortisol measurements cannot reflect changes in the daily pattern of cortisol levels. De Jong et al. [54] have shown that pigs housed under barren condition show a blunted circadian rhythm in cortisol compared to pigs housed in enriched conditions and that a blunted rhythm may indicate decreased welfare. Moreover, it has been shown that CBG (corticosteroid-binding globulin) concentration can decrease under stressful conditions [55], making conclusions based only on plasma cortisol levels a complex task. Thus, more detailed investigation on circadian rhythm and the free (= active) vs. bound (= inactive) ratio of cortisol is needed to draw comprehensive conclusions regarding the effect of housing conditions and gender on hypothalamic–pituitary axis (HPA) activity.

On the other hand, behavioral observations in our study revealed that mixed animals more often showed severe agonistic behavior and were thus probably more stressed than animals from the standard or enriched housing environments [56]. Moreover, the poorer growth performance of mixed pigs in the present study can also be taken as an indicator of stressful housing. Studies by Ekkel et al. [57] showed that housing with social mixing of pigs has a negative impact on growth performance and on welfare when compared to “specific stress-free” housing environments. These results are also consistent with those from other reports on the effect of mixing on ADG [30,31,32]. Here, it is important to note that, although mixing stress most likely caused higher stress levels in immunocastrates as well, the intensity of the stressor ‘mixing’ stressor was not sufficient to negatively affect antibody response to GnRH or even to cause non-responders. 

In the present study, the growth performance of immunocastrates was significantly higher after the second vaccination than that of boars and barrows, which agrees with the meta-analysis of literature reports [46]. A study by Pauly et al. [58], however, had shown that after the second vaccination, only differences between immunocastrates and boars remain, but not between immunocastrates and barrows. Until the second vaccination is applied, the growth performance of immunocastrates is identical to that of boars [46,58]. Over the entire fattening period of the present experiment, no differences in growth performance between the sex groups occurred.However, this does not exclude the possibility, that immunocastrates could be superior to barrows and boars over the entire fattening period. The extent to which the growth performance after the second vaccination or throughout the entire fattening period differs between the sex groups mainly depends on the genetically determined level of feed consumption of a genotype [33] and on the timing of the second vaccination [13]. The economic relevance of the higher growth rates of immunocastrates after the second immunization against GnRH gains relevance only if the feed conversion ratio is also competitive and the fattening duration decreases compared to barrows and boars [33]. 

## 5. Conclusions

This study shows that immunocastration is a reliable technique to supresses testicular functions under different housing conditions. Regardless of housing conditions, testosterone concentrations drop after the second Improvac^®^-vaccination to a low level, comparable to barrows. Furthermore, all carcasses of immunocastrates were free of boar taint, whereas a considerable number of boar carcasses were affected by boar taint. Reproductive organs, as well, react to the vaccinations and result in lower weights than in boars. In our study, based on Pietrain x German Landrace and two vaccinations at the age of 12 and 22 weeks, the growth performance between sex groups did not differ throughout the total fattening period. On the other hand, immunocastrates had a higher growth performance after the second vaccination which can be used in economic terms by choosing the optimal time for the second vaccination. Mixing had been applied as a standardized method to increase stress. A negative influence on growth performance by this was obvious and points to a moderate stress. However, this social stressor did not modify the immune response upon vaccination against GnRH or cause any non-responders in our study.

## Figures and Tables

**Table 1:** Genital tract weight (g) of boars, immunocastrates and barrows (*n* = 48 per sex group) housed under three different housing conditions (enriched—*n* =12, standard—*n* = 12 and mixing—*n* = 24).

Parameter	Boars (*n* = 48)	Immunocastrates (*n* = 48)	Barrows (*n* = 48)	*p*-Value	Enriched (*n* = 36)	Standard (*n* = 36)	Mixing (*n* = 72)	*p*-Value
Testes *	722.57 ± 21.43 ^b^	288.41 ± 20.77 ^a^	-	*<.0001*	490.97 ± 26.65	521.02 ± 24.23	504.48 ± 23.91	*0.7001*
Vesicular gl.	274.15 ± 16.26 ^b^	38.94 ± 15.59 ^a^	-	*<.0001*	140.04 ± 19.89	175.95 ± 19.15	153.63 ± 15.79	*0.3026*
Bulbourethral gl.	158.36 ± 10.87 ^b^	58.95 ± 10.87 ^a^	-	*<.0001*	105.42 ± 11.51	115.65 ± 11.37	104.90 ± 10.67	*0.3930*
Prostate	9.24 ± 0.47 ^b^	3.38 ± 0.46 ^a^	-	*<.0001*	6.18 ± 0.53	6.85 ± 0.53	5.89 ± 0.50	*0.4648*
Urogenital tract	540.61 ± 20.46 ^c^	214.01 ± 18.16 ^b^	115.41 ± 17.85 ^a^	*<.0001*	281.00 ± 18.63	310.67 ± 18.68	278.36 ± 15.98	*0.2729*

Parameters within a row with different superscripts differ significantly (*p* < 0.05); * both testes weighed with epididymes.

**Table 2:** Androstenone and skatole concentrations in boars, immunocastrates and barrows (*n* = 48 per sex group) housed under three different housing conditions (enriched—*n* = 12, standard—*n* = 12 and mixing—*n* = 24).

Parameter	Boars (*n* = 48)	Immunocastrates (*n* = 48)	Barrows (*n* = 48)	*p*-Value	Enriched (*n* = 36)	Standard (*n* = 36)	Mixing (*n* = 72)	*p*-Value
Androstenone	2.53 ± 0.50 ^b^	<0.24 ^a^	-	<.0001	0.64 ± 0.15	0.67 ± 0.15	0.74 ± 0.14	0.7184
Skatole	0.037 ± 0.005 ^b^	0.020 ± 0.003 ^a^	0.021 ± 0.003^a^	<.001	0.021 ± 0.003	0.029 ± 0.005	0.025 ± 0.003	0.1179

Parameters within a row with different superscripts differ significantly (*p* < 0.05). All immunocastrates had androstenone levels below the limit of detection (<0.24 µg/g fat). Androstenone is testis-derived and was not analyzed in barrows.

**Table 3:** Cortisol levels in boars, immunocastrates and barrows (*n* = 48 per sex group) housed under three different housing conditions (enriched—*n* = 12, standard—*n* = 12 and mixing—*n* = 24).

Parameter	Boars (*n* = 48)	Immunocastrates (*n* = 48)	Barrows (*n* = 48)	*p*-Value	Enriched (*n* = 36)	Standard (*n* = 36)	Mixing (*n* = 72)	*p*-Value
Cortisol - B1	27.96 ± 2.77	26.37 ± 2.75	28.93 ± 2.74	*0.7025*	31.76 ± 2.94	26.34 ± 2.87	25.16 ± 2.49	*0.1353*
Cortisol - B2	23.82 ± 3.04	21.37 ± 3.03	27.31 ± 3.03	*0.0584*	23.44 ± 3.19	23.82 ± 3.13	25.23 ± 2.81	*0.7137*
Cortisol - B3	19.49 ± 1.47	18.56 ± 1.40	17.77 ± 1.34	*0.6867*	17.63 ± 1.46	21.49 ± 1.78	16.97 ± 0.99	*0.0629*
Cortisol - B4	59.15 ± 4.41 ^b^	45.29 ± 4.36 ^a^	49.74 ± 4.34 ^ab^	*0.0049*	53.57 ± 4.66	48.26 ± 4.61	52.36 ± 3.86	*0.4735*

Blood Samples—B1: before V1, B2: before V2, B3: 2 weeks after V2, B4 at slaughter; parameters within a row with different superscripts differ significantly (*p* < 0.05).

**Table 4:** Growth performance (average daily gain, ADG, in g) in boars, immunocastrates and barrows (*n* = 48 per sex group housed under three different housing conditions (enriched—*n* = 12, standard—*n* = 12 and mixing—*n* = 24).

Parameter	Boars (*n* = 48)	Immunocastrates (*n* = 48)	Barrows (*n* = 48)	*p*-Value	Enriched (*n* = 36)	Standard (*n* = 36)	Mixing (*n* = 72)	*p*-Value
ADG - Phase 1	854 ± 23	864 ± 22	911 ± 22	*0.0417*	862 ± 23	887 ± 23	880 ± 21	*0.4747*
ADG - Phase 2	923 ± 46	905 ± 46	963 ± 46	*0.0625*	969 ± 47^b^	926 ± 47 ^ab^	894 ± 44 ^a^	*0.0099*
ADG - Phase 3	869 ± 20 ^b^	967 ± 20 ^c^	816 ± 20 ^a^	*<.0001*	911 ± 21 ^b^	882 ± 21^ab^	859 ± 18^a^	*0.0438*
ADG - Total Fattening	855 ± 20	906 ± 20	879 ± 20	*0.0694*	898 ± 21	885 ± 21	886 ± 19	*0.1220*

Feeding phases: Phase 1—at an age of 10 to 18 weeks; Phase 2: at an age of 18 to 22 weeks; Phase 3—at an age of 22 to 27/28 weeks; Total fattening period: ADG (g) of total fattening period (age of 10 to 27/28 weeks); parameters within a row with different superscripts differ significantly (*p* < 0.05).
